# Transcriptional Regulation by the Velvet Protein VE-1 during Asexual Development in the Fungus Neurospora crassa

**DOI:** 10.1128/mbio.01505-22

**Published:** 2022-08-01

**Authors:** Sara Cea-Sánchez, María Corrochano-Luque, Gabriel Gutiérrez, N. Louise Glass, David Cánovas, Luis M. Corrochano

**Affiliations:** a Departamento de Genética, Facultad de Biología, Universidad de Sevilla, Seville, Spain; b Plant and Microbial Biology Department, University of California, Berkeley, Berkeley, California, USA; Karlsruhe Institute of Technology (KIT)

**Keywords:** conidiation, light, RNA-seq, velvet complex, transcriptional regulation

## Abstract

Asexual reproduction in fungi facilitates the dispersal and colonization of new substrates and, in pathogenic fungi, allows infection of plants and animals. The velvet complex is a fungus-specific protein complex that participates in the regulation of gene expression in response to environmental signals like light, as well as developmental processes, pathogenesis, and secondary metabolism. The velvet complex in the fungus Neurospora crassa is composed of three proteins, VE-1, VE-2, and LAE-1. Mutations in *ve-1* or *ve-2*, but not in *lae-1*, led to shorter heights of aerial tissue, a mixture of aerial hyphae and developing macroconidia, and increased microconidiation when they were combined with mutations in the transcription factor gene *fl*. VE-2 and LAE-1 were detected during vegetative growth and conidiation, unlike VE-1, which was mostly observed in samples obtained from submerged vegetative hyphae. We propose that VE-1 is the limiting component of the velvet complex during conidiation and has a major role in the transcriptional regulation of conidiation. Characterization of the role of VE-1 during mycelial growth and asexual development (conidiation) by transcriptome sequencing (RNA-seq) experiments allowed the identification of a set of genes regulated by VE-1 that participate in the regulation of conidiation, most notably the transcription factor genes *vib-1* and *fl*. We propose that VE-1 and VE-2 regulate the development of aerial tissue and the balance between macro- and microconidiation in coordination with FL and VIB-1.

## INTRODUCTION

Asexual reproduction in fungi facilitates the dispersal and colonization of new substrates and is a key step in the infection of plants and animals by pathogenic fungi. Although the formation of vegetative spores or conidia is a conserved fungal trait, the molecular mechanisms and the regulatory genes that participate in asexual development show differences among fungi (1–7). Two types of vegetative spores, or conidia, have been described in the ascomycete Neurospora crassa: macroconidia and microconidia. Macroconidia are large and multinucleate, while microconidia are smaller and uninucleate. Macroconidia are produced abundantly under most growth conditions, but microconidia are only observed under specific growth conditions, including high humidity, low temperature, and limited nutrients (8, 9). We will use the terms macroconidia and conidia interchangeably, as they are the most easily observed and most frequently used type of N. crassa conidia.

Macroconidiation is usually induced by desiccation, through the transfer of cultures from liquid to an air interface, and a reduction of nutrients. In addition, macroconidiation is regulated by light, the levels of CO_2_, and the circadian clock (5, 8). During macroconidiation, vegetative hyphae lift up from the surface substrate and develop a mass of aerial hyphae that leads to the development of a string of conidia held together by connective threads until they are dispersed by air currents (10). Single microconidia, on the contrary, are produced by budding of vegetative hyphae and have been proposed to serve as male fertilization cells during the sexual cycle (9). Mutations that block macroconidiation do not modify microconidiation, although conidiation (*con*) genes isolated by their expression during macroconidiation are also detected in microconidia (11). Several genes that participate in the regulation of macroconidiation have been identified by mutations that block this developmental pathway. A key regulatory gene is *fl*, which encodes a Zn_2_Cys_6_ binuclear zinc cluster domain transcription factor (TF) (12). Overexpression of *fl* in vegetative mycelia leads to macroconidiation in submerged cultures, and FL binding sites have been identified in genes regulated by FL, such as the gene *eas*, encoding the hydrophobin that coats macroconidia (13, 14). The gene *fl* is induced by light in a process regulated by binding of the light-dependent transcription factor complex white collar complex (WCC) to the promoter. It has been proposed that the activation of *fl* by light is a key element in the regulation by light of macroconidiation (15).

The velvet complex is a fungus-specific protein complex that participates in the regulation of conidiation in many fungi (16, 17). The activity and regulation of the velvet complex have been described in detail in Aspergillus nidulans. The A. nidulans velvet complex is composed of three proteins, VeA, VelB, and LaeA (18). VeA and VelB contain a 150-amino-acid (aa) velvet domain that participates in protein-protein interactions and DNA binding. The structure of the velvet domain resembles the DNA-binding fold of the mammalian transcription factor NF-κB (19). It has been proposed that the velvet complex regulates transcription by DNA binding to promoters and chromatin modification (20). In A. nidulans, the components of the velvet complex are observed in the cytoplasm and nuclei. VeA and VelB interact with each other, and VeA also interacts with the importin KapA to enter the nucleus in a process that is regulated by light (21). The third component of the velvet complex is LaeA, a methyltransferase that is required for the regulation of secondary metabolism (18, 22). Other methyltransferases, kinases, and photoreceptors interact with components of the velvet complex, presumably to regulate its activity (20, 23, 24). In addition, VosA, which also contains a velvet domain, interacts with VelB to regulate spore maturation and viability in A. nidulans (25, 26). The velvet gene family also includes a fourth member, *ve-3*/*velC*, which belongs to a nonmonophyletic group of velvet genes (6). In A. nidulans, VelC is a negative regulator of conidiation and a positive regulator of sexual development (27), while in N. crassa, VE-3 appears to have a truncated velvet domain and is not a component of the velvet complex (28).

The velvet complex in N. crassa is composed of VE-1, VE-2, and LAE-1. They interact in vegetative hyphae to form a complex that participates in the regulation of sexual and asexual development, secondary metabolism, and the accumulation of carotenoid pigments (28). Strains with mutations in *ve-1*, *ve-2*, or *lae-1* showed reduced sexual development, but alterations in asexual development (shortened aerial hyphae and increased conidiation) were observed in *ve-1* or *ve-2* mutant strains only (28). Mutations in the genes encoding components of the velvet complex resulted in changes in the accumulation of secondary metabolites, including the siderophore coprogen, and of carotenoids. Transcriptome sequencing (RNA-seq) analysis performed in liquid cultures under growth conditions that promoted the accumulation of secondary metabolites identified a set of about 1,500 genes regulated by VE-1 (28). Heterologous expression of *ve-1* and *ve-2* complemented the phenotypes of A. nidulans mutants with mutation of *veA* or *velB*, respectively. Similarly, the expression of A. nidulans *veA* in the N. crassa *ve-1* mutant restored the wild-type phenotype (28).

VE-1 has been observed in the cytoplasm and nuclei of vegetative hyphae, and the nuclear accumulation of VE-1 increases during conidiation. In addition, VE-1 is degraded by the proteasome during conidiation, but the degradation of VE-1 is regulated by light, presumably to allow a quick transcriptional response to changes in light intensity during conidiation (29). It is not known how VE-1 and the other components of the velvet complex interact and regulate transcription during conidiation. The other velvet protein, VOS-1, was found to interact with VE-1 and VE-2, but no phenotype in conidial development was found in N. crassa (28).

Here, we characterized the role of VE-1 in transcriptional regulation during mycelial growth and during conidiation by RNA-seq experiments performed with cultures kept in dark or exposed to light. We propose that VE-1 is the limiting component of the velvet complex during conidiation and that it has a major role in the transcriptional regulation of the developmental process of conidiation.

## RESULTS

### The *ve-1* and *ve-2* genes are required for aerial growth and conidiation.

**FIG 1:**
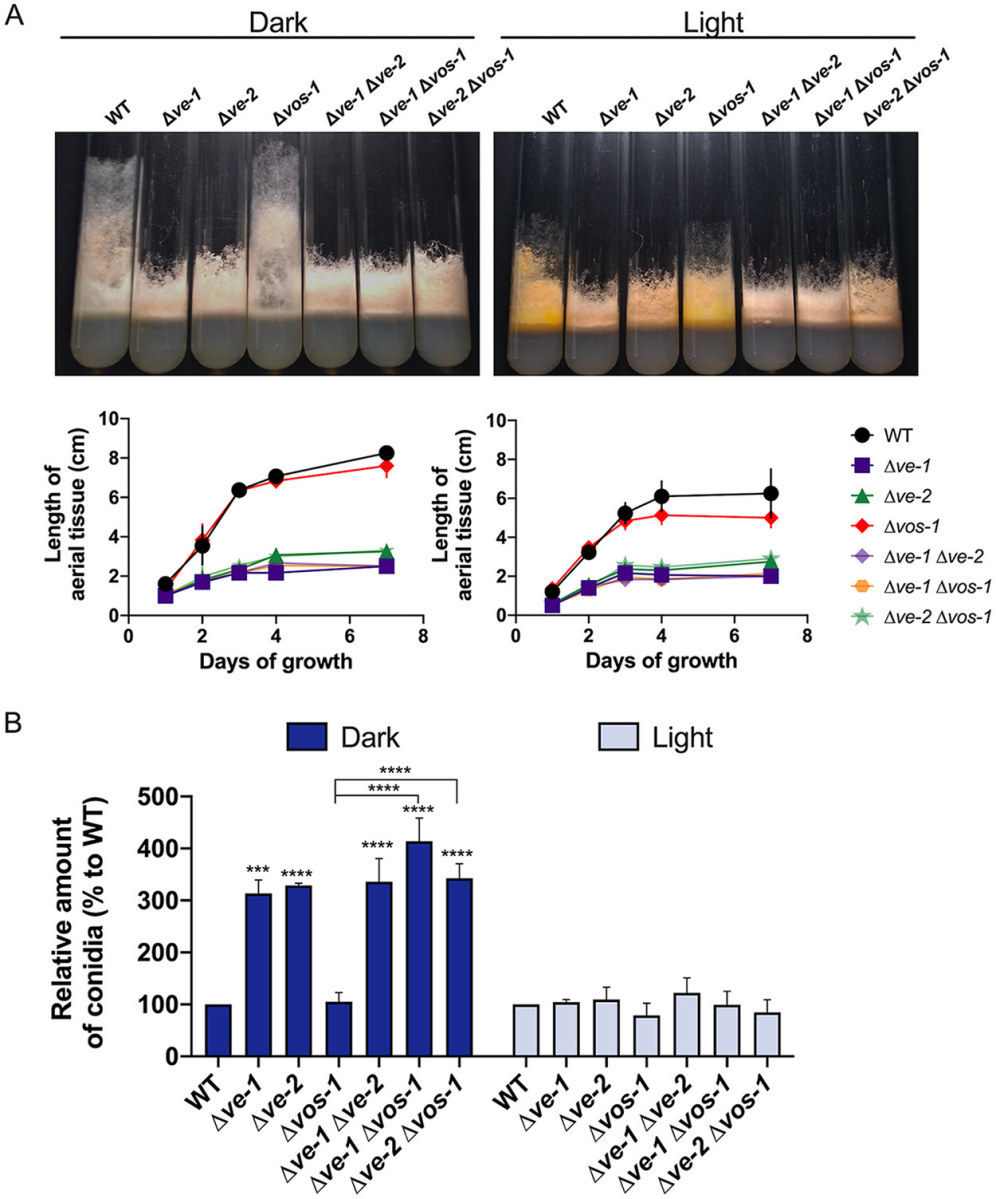
Mutants with mutations in *ve-1* and *ve-2* have defects in aerial tissue and increased conidiation. (A) Photographs of growth tube phenotypes of the wild type, Δ*ve-1*, Δ*ve-2*, and Δ*vos-1* mutants, and double deletion mutants after 3 days of growth at 30°C in the dark or the light. Graphs show the lengths of aerial hyphae over time in the wild type and the mutants in the light or the dark. (B) Relative amounts of conidia produced by the wild type and single and double deletion mutants. Conidia were collected from cultures grown in the dark and the light at 30°C for 3 days, and the amounts plotted relative to the amount produced by the wild type in the dark or the light. The wild-type strain produced 1.5 × 10^8^ conidia/tube in the dark compared to 4.7 × 10^8^ conidia/tube in the light. The plots show the mean values and standard errors from three independent experiments. Two-way analysis of variance (ANOVA) comparisons were performed between the values for all strains. Statistical significance is shown only for comparisons between the wild-type and single mutant strains and between every combination of a single mutant with the corresponding double mutant strains (Δ*ve-1* versus Δ*ve-1* Δ*ve-2*, Δ*ve-1* versus Δ*ve-1* Δ*vos-1*, etc.). Significant differences are indicated by asterisks, as follows: *, *P* < 0.05; **, *P* < 0.01; ***, *P* < 0.001; ****, *P* < 0.0001.

Initial studies have shown that the absence of VE-1 led to a light-dependent shortening of aerial tissue, a mixture of aerial hyphae and developing macroconidia, that required the activity of the photoreceptor WC-1 (29). This observation, together with the proposed role of the velvet complex in the regulation of asexual development in N. crassa (28), prompted us to further characterize the roles of VE-1 and VE-2 in the growth of aerial tissue, the amounts of conidia in single and double deletion mutants, and their possible regulation by light. Aerial tissue is defined by the growth of conidiating hyphae away from the supporting mycelia in glass tubes with solid agar medium. We observed a 25% reduction in the length of aerial hyphae in the wild-type strain grown under illumination. Shorter aerial hyphae were observed in Δ*ve-1* and Δ*ve-2* mutant strains than in the wild type both in the dark and the light (Fig. 1A). A reduction in the growth of aerial tissue has been reported for a strain with reduced adenylyl cyclase activity, a *cr-1* mutant. The growth of aerial tissue in this mutant was restored after the addition of cAMP to the growth medium (30), but supplementation with cAMP did not restore the growth of aerial tissue in Δ*ve-1* or Δ*ve-2* mutant strains (Fig. S1A in the supplemental material). In the wild-type strain, conidiation was increased 3-fold by light exposure. In addition, Δ*ve-1* and Δ*ve-2* mutant strains displayed 3.2- and 3.3-fold higher conidiation levels, respectively, but only in cultures kept in the dark (Fig. 1B). The Δ*vos-1* mutant did not show any alteration in the growth of aerial tissue or conidial production and behaved like the wild-type strain under all conditions. Deletion of *vos-1* did not alter the phenotypes of either the Δ*ve-1* or the Δ*ve-2* mutant strain, and the double Δ*ve-1* Δ*ve-2* mutant displayed the same short-aerial-tissue phenotype and increase in conidiation in the dark (3.5-fold higher than the wild type) as the corresponding single mutants (Fig. 1). Deletion of *ve-2*, *vos-1*, or both did not affect the viability of conidia in N. crassa, unlike in A. nidulans (Fig. S1B) (25, 26). Our results support the proposal that the absence of either VE-1 or VE-2 disrupts the activity of the velvet complex, resulting in alterations in the growth of aerial tissue and of conidiation in the dark.

10.1128/mbio.01505-22.1 FIG S1 Growth tube phenotypes and viability of conidia. (A) Photographs of culture tube phenotypes of wild type and mutants with cAMP supplementation. The graph on the right shows aerial tissue lengths in the wild-type, Δ*ve-1*, Δ*ve-2*, and Δ*ve-1* Δ*ve-2* strains after 5 days of growth at 30°C in the light with 2.5 mM cAMP, presented as the mean values and standard errors from three independent experiments. (B) Conidial samples from the wild type and the Δ*ve-2*, Δ*vos-1*, and Δ*ve-2* Δ*vos-1* mutant strains were collected and stored at 4°C for 0 to 21 days. Conidial viability was assayed by plating diluted samples and assessing the number of colonies after 2 days of growth at 34°C from each sample associated with the period of storage at 4°C. The plot shows the viability of conidia stored at 4°C relative to the viability observed at the time of collection from each strain as the average value and standard error from three experiments. (C) Photographs of culture tube phenotypes of wild-type, Δ*ve-1*, Δ*ve-2*, Δ*lae-1*, *ve-1^FLAG^*, *ve-2^FLAG^*, and *lae-1^FLAG^* strains after 3 days of growth at 30°C in the light. The graph on the right shows the lengths of aerial tissue of the wild-type and other strains, presented as the mean values and standard errors from three independent experiments. Two-way ANOVA: *, *P* < 0.05; **, *P* < 0.01; ***, *P* < 0.001; ****, *P* < 0.0001. Download FIG S1, JPG file, 0.3 MB. Copyright © 2022 Cea-Sánchez et al. 2022 Cea-Sánchez et al. https://creativecommons.org/licenses/by/4.0/ This content is distributed under the terms of the Creative Commons Attribution 4.0 International license.

### VE-1 and VE-2 localize to nuclei and cytoplasm of aerial tissue.

**FIG 2:**
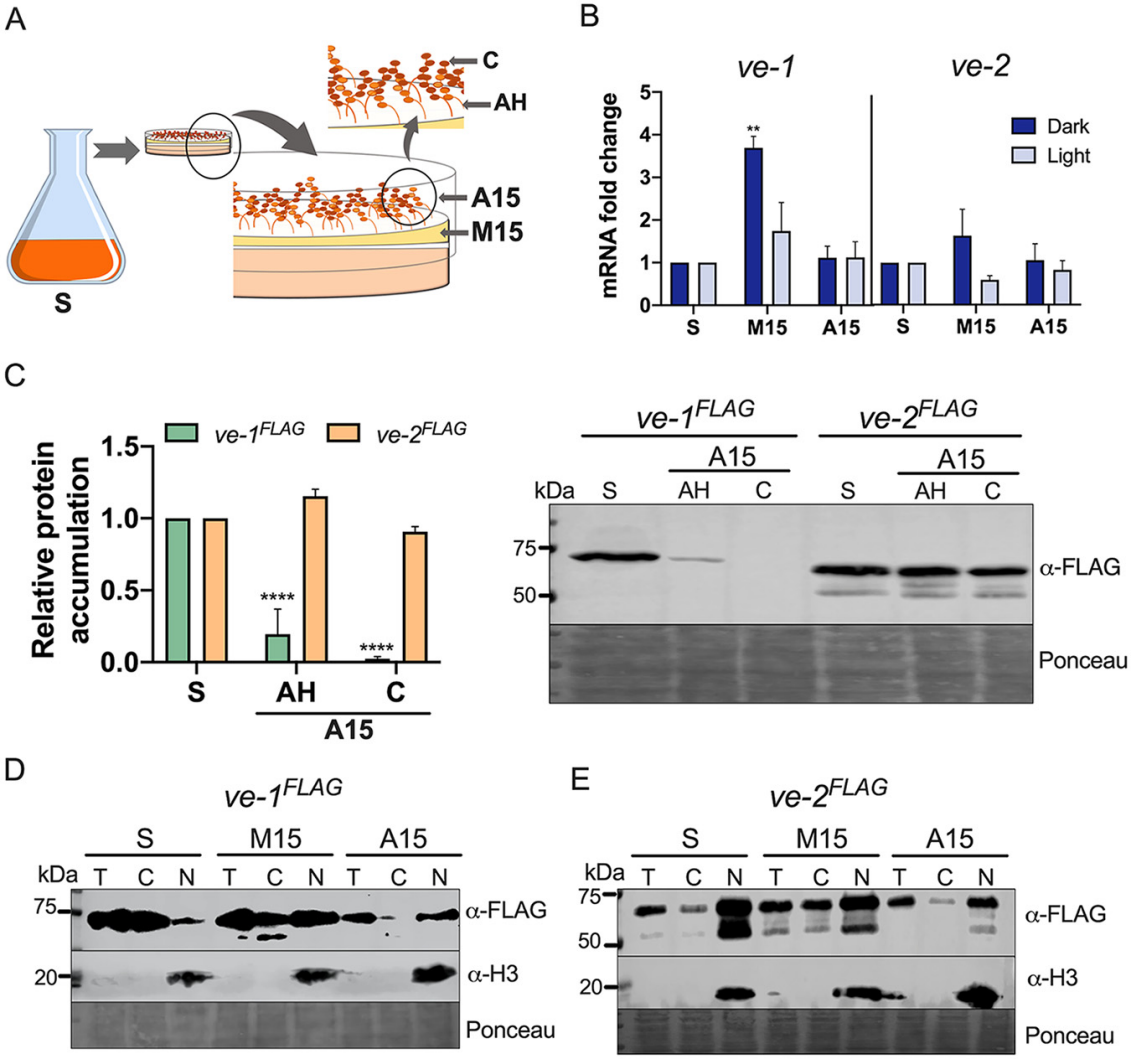
Accumulation and subcellular localization of VE-1 and VE-2 during asexual development. (A) Samples from cultures kept in the dark or the light were obtained from mycelia growing vegetatively in submerged liquid medium for 24 h (submerged vegetative mycelia [S]), from supporting vegetative mycelia after 15 h of induction of conidiation (M15), and from aerial tissue after 15 h of induction of conidiation (A15). Aerial hyphae (AH) and conidia (C) were separated from aerial tissue (A15) by centrifugation. (B) Relative levels of accumulation of *ve-1* and *ve-2* mRNAs during asexual development. Quantitative RT-PCR experiments were performed to measure relative levels of accumulation of mRNAs in three different stages of development. The results were normalized to the corresponding *tub-2* mRNA accumulation to correct for sampling errors. Then, the results were normalized to those obtained with submerged vegetative mycelia growing in liquid medium for 24 h (S). (C) Accumulation of VE-1 and VE-2 proteins during conidiation in the light. We used the *ve-1^FLAG^* and *ve-2^FLAG^* strains. Proteins were separated by SDS-PAGE and hybridized with an antibody specific for FLAG. An amount of 70 μg of protein was loaded per lane. As a loading control, we used Ponceau staining of each protein sample. (D) Subcellular localization of VE-1 during conidiation in the dark. We used the *ve-1^FLAG^* strain. Total protein samples (T) or samples enriched in cytoplasmic (C) or nuclear (N) proteins were separated by SDS-PAGE and hybridized with antibodies specific for FLAG or histone H3. An amount of 80 to 120 μg of protein was loaded per lane. As a loading control, we used Ponceau staining of each protein sample. (E) Subcellular localization of VE-2 during conidiation in the light. We used the *ve-2^FLAG^* strain. Total protein samples (T) or samples enriched in cytoplasmic (C) or nuclear (N) proteins were separated by SDS-PAGE and hybridized with antibodies specific for FLAG or histone H3. An amount of 70 μg of protein was loaded per lane. As a loading control, we used Ponceau staining of each protein sample. Plots show the mean values and standard errors of three independent experiments. Two-way ANOVA: *, *P* < 0.05; **, *P* < 0.01; ***, *P* < 0.001; ****, *P* < 0.0001.

The role of the velvet complex in asexual development suggested that it could be present in the nuclei of aerial tissue to regulate transcription. We have previously reported the presence of the velvet complex in the nuclei and cytoplasm of vegetative hyphae (28) and the accumulation of VE-1 in the nuclei and cytoplasm of aerial tissue in a light-regulated manner (29). To characterize the accumulation of mRNAs and proteins during conidiation, we collected samples from vegetative mycelia cultured in liquid medium (submerged vegetative mycelia) prior to the induction of conidiation. To induce conidiation, we transferred vegetative mycelia to the surface of a solid medium plate. Fifteen hours after the induction of conidiation, we collected samples from the supporting vegetative mycelia (M15) and from aerial tissue (A15) for RNA and protein quantification. In some cases, we further separated mature conidia (C) from aerial hyphae (AH) to characterize the protein content from each developmental structure (Fig. 2A). For RNA quantification, we used the wild-type strain, and for protein quantification, we employed strains with alleles of *ve-1* or *ve-2* fused to the coding sequence of the FLAG epitope to allow immunodetection by Western blotting. Each new allele, *ve-1^FLAG^* and *ve-2^FLAG^*, replaced each wild-type allele and was under the control of the original promoter. The growth of strains with the *ve-1^FLAG^* or *ve-2^FLAG^* alleles was similar to that of the wild-type strain (Fig. S1C).

The *ve-1* and *ve-2* mRNAs were observed in vegetative mycelia (both submerged vegetative mycelia [S] and supporting vegetative mycelia [M15]) and aerial tissue (A15). We did not detect any major difference between these developmental stages, regardless of the light condition, except for an increase in the amount of *ve-1* mRNA in supporting vegetative mycelia in the dark (Fig. 2B). We confirmed the detection of VE-1 and VE-2 in submerged vegetative mycelial samples, but the amounts of VE-1 in aerial hyphae and conidia were reduced, unlike VE-2, which accumulated in similar amounts in submerged vegetative mycelia, aerial hyphae, and conidia (Fig. 2C). Light exposure did not modify the pattern of VE-2 accumulation (Fig. S2).

10.1128/mbio.01505-22.2 FIG S2 Accumulation of VE-1, VE-2, and LAE-1 during conidiation. Protein samples were obtained from submerged vegetative mycelia (S) or supporting vegetative mycelia 15 h (M15) or 24 h (M24) following transfer from liquid medium to the surface of an agar plate with minimal medium to induce conidiation. Aerial tissue was collected after 15 h (A15) or 24 h (A24) of induction of conidiation. Cultures were kept in the dark or the light or grown in the dark and exposed to 30 min of light prior to collection of aerial tissue. We used the *ve-1^FLAG^*, *ve-2^FLAG^*, and *lae-1^FLAG^* strains. Proteins were separated by SDS-PAGE and hybridized with an antibody specific for FLAG. An amount of 70 μg of proteins was loaded per lane. As a loading control, we used Ponceau staining of each protein sample. Download FIG S2, JPG file, 0.4 MB. Copyright © 2022 Cea-Sánchez et al. 2022 Cea-Sánchez et al. https://creativecommons.org/licenses/by/4.0/ This content is distributed under the terms of the Creative Commons Attribution 4.0 International license.

VE-1 was detected in the cytoplasmic and nuclear fractions in protein samples obtained from vegetative mycelia (submerged and supporting vegetative mycelia) and aerial tissue, despite the low abundance of this protein in aerial tissue (Fig. 2D). We observed more VE-2 in nuclear than in cytoplasmic fractions in the light (Fig. 2E), but the accumulation of VE-1 in nuclear fractions was mostly observed after the induction of conidiation in the dark (Fig. 2D), as we previously reported in light-exposed cultures (29). Our observations on the abundances and the nuclear localization of VE-1 and VE-2 suggest that VE-1 is the limiting component of the velvet complex and that the regulation of its abundance and subcellular localization plays a key role in the activity of the velvet complex during asexual development.

### Transcriptomic comparisons identified genes regulated by VE-1.

**FIG 3:**
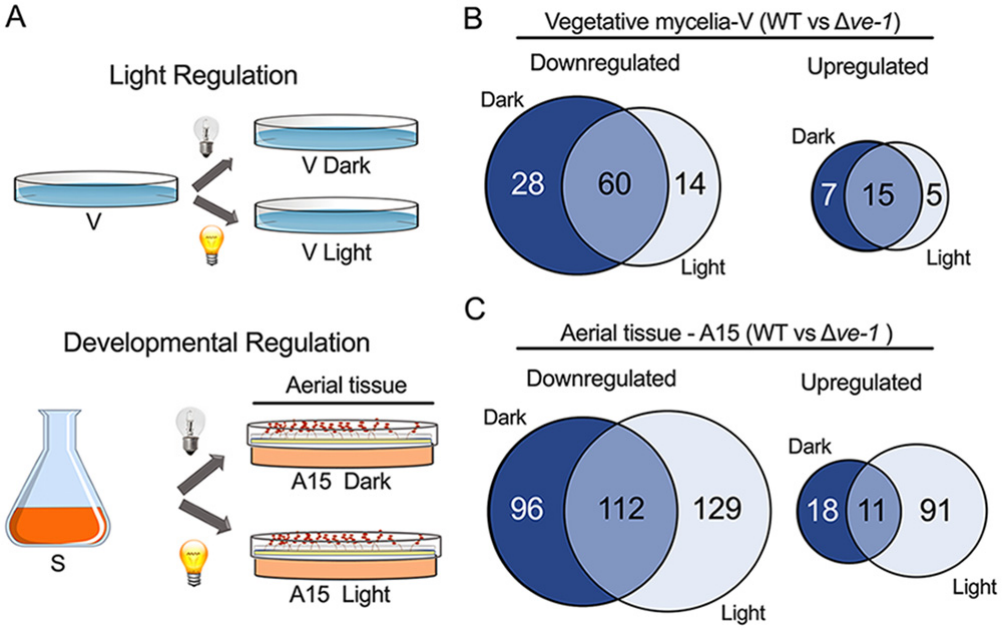
Comparison of transcriptional regulation during vegetative growth and conidiation in response to light. (A) Schematic representation of the experimental conditions for the characterization of the light-dependent transcriptome during vegetative growth (top) and the transcriptome during asexual development (bottom). For the characterization of the light-dependent transcriptome during vegetative growth (Light Regulation), submerged vegetative mycelia of the wild-type and Δ*ve-1* strains were grown in liquid medium for 48 h at 22°C (V) and then exposed to light (30 min) (V Light) or kept in the dark as a control (V Dark). For the characterization of the transcriptome during asexual development (Developmental Regulation), submerged vegetative mycelia were grown at 30°C for 24 h in liquid medium and transferred to the surface of a plate containing solid minimal medium to induce asexual development. The aerial tissue (containing aerial hyphae and developing conidia) was collected 15 h after induction of conidiation. The aerial tissue was exposed to light (30 min) prior to sample collection (A15 Light) or kept continuously in the dark (A15 Dark). (B, C) Transcriptional regulation by VE-1. (B) Venn diagrams with the numbers of genes with differential levels of mRNA accumulation in vegetative mycelia of the Δ*ve-1* mutant strain compared to those of the wild-type strain in the dark or after exposure to 30 min of light. (C) Venn diagrams with the numbers of genes with differential levels of mRNA accumulation in aerial tissue of the Δ*ve-1* mutant strain compared to that of the wild-type strain in the dark or after exposure to 30 min of light.

The alterations in aerial hyphal growth and conidiation observed in the Δ*ve-1* mutant, together with our previous proposal that VE-1 is required for full light-dependent transcription along with the WCC (29), prompted us to characterize the role of VE-1 in transcription using RNA-seq experiments. To characterize the transcriptional response to light in vegetative mycelia, cultures of the wild-type and Δ*ve-1* strains were grown in liquid medium for 47.5 h in the dark, followed by 30 min of growth in the light or being kept in the dark as a control, prior to mRNA purification from vegetative mycelia (light regulation experiments). Under these conditions, mycelia grew vegetatively and did not form conidiophores. To characterize the transcriptional response to conidiation, the wild-type and Δ*ve-1* strains were grown in liquid medium for 24 h in the dark, transferred to solid medium to induce conidiation, and further incubated for 15 h in the dark or for 14.5 h in the dark followed by 30 min of exposure to the light prior to mRNA purification from aerial tissue (developmental regulation experiments) (Fig. 3A). We considered differentially expressed genes (DEGs) to be those showing differences in mRNA accumulation with a log_2_ fold change of 2 or more (log_2_FC ≥ 2).

To identify genes that changed their mRNA accumulation levels in the Δ*ve-1* mutant strain regardless of their regulation by conidiation and/or light, we made direct comparisons of mRNA accumulation levels in the wild-type and Δ*ve-1* strains for all expressed genes under all conditions.

In vegetative mycelia, we identified 129 genes with changes in mRNA accumulation (102 downregulated and 27 upregulated) (Fig. 3B and Data Set S1). The transcriptional effect of the Δ*ve-1* mutation during conidiation was larger: 457 genes had changes in mRNA accumulation, about 5% of the N. crassa genome (Fig. 3B and Data Set S1). We identified 237 misregulated genes during conidiation in the dark (208 genes downregulated and 29 upregulated). Exposure to light during conidiation increased the number of misregulated genes to 343 genes (241 downregulated and 102 upregulated). However, only 123 genes were misregulated during conidiation under both conditions, indicating that there was a large set of genes that showed changes in mRNA accumulation in the Δ*ve-1* mutant when cultures were kept either in the dark or in the light (Fig. 3C). This observation supported the proposal of a major role for VE-1 during transcriptional regulation of conidiation in the dark and the light. Among the genes misregulated in the Δ*ve-1* mutant during conidiation, we identified seven transcription factor genes, including the *vos-1* velvet gene and *vib-1*, and eight genes for methyltransferases (Data Set S1).

10.1128/mbio.01505-22.6 DATA SET S1 Genes regulated by VE-1 in Neurospora crassa. Download Data Set S1, XLSX file, 2.9 MB. Copyright © 2022 Cea-Sánchez et al. 2022 Cea-Sánchez et al. https://creativecommons.org/licenses/by/4.0/ This content is distributed under the terms of the Creative Commons Attribution 4.0 International license.

### Comparative transcriptomics revealed a role of VE-1 in transcriptional regulation during asexual development but not during vegetative growth.

To identify additional changes in the transcriptome due to the absence of VE-1, we compared the transcriptomes of the wild-type and Δ*ve-1* strains during the light response in vegetative mycelia and during asexual development (Fig. 3A). The light-dependent transcriptome in vegetative mycelia of the wild type was very similar to that in the Δ*ve-1* mutant strain, comprising 128 genes in the wild-type strain and 97 genes in the Δ*ve-1* mutant strain, with a large overlap (90 genes) that included genes that had been characterized as light regulated in N. crassa, e.g., *con* (conidiation) genes and genes required for the biosynthesis of carotenoids, DNA repair (photolyase and UV endonuclease), and protection from oxidative stress (catalase and peroxidase) (Fig. S3A and Data Set S2). The light-dependent transcriptome in aerial tissue was smaller than that in vegetative mycelia. We identified 74 and 31 light-regulated genes in the wild-type and the Δ*ve-1* mutant strain, respectively. However, the small overlap between the two transcriptomes (11 genes) suggested a role for VE-1 in transcriptional regulation by light in aerial tissue (Fig. S3A and Data Set S2).

10.1128/mbio.01505-22.3 FIG S3 Comparison of the transcriptional response to conidiation between the wild-type and the Δ*ve-1* mutant strains. (A) Venn diagrams with the numbers of genes differentially regulated (up- or downregulated) by light in vegetative mycelia (left) or aerial tissue (right) in the wild-type and the Δ*ve-1* mutant strains. (B) Venn diagrams with the numbers of genes differentially regulated (up- or downregulated) during conidiation in the dark or after exposure to light (30 min) in the wild-type and the Δ*ve-1* mutant strains. (C) Box plot summarizing the fold changes (log_2_) for the 477 differentially expressed genes identified in all conditions and strains. The upper panel shows the upregulated genes (*n* = 358), and the bottom panel shows the downregulated genes (*n* = 119). (D) Heat map of relative transcript levels of genes encoding transcription factors in submerged vegetative mycelia (S) and in aerial tissue after 15 h of induction of conidiation (A15) in the wild-type and Δ*ve-1* mutant strains. Alterations in the development of conidia (asexual phenotype) are indicated by alterations in the growth of aerial tissue (A) or in the production of conidia (C). Genes were clustered and manually grouped by the effect of the Δ*ve-1* mutation on relative transcript levels. Download FIG S3, JPG file, 0.8 MB. Copyright © 2022 Cea-Sánchez et al. 2022 Cea-Sánchez et al. https://creativecommons.org/licenses/by/4.0/ This content is distributed under the terms of the Creative Commons Attribution 4.0 International license.

10.1128/mbio.01505-22.7 DATA SET S2 Characterization of the regulation of transcription by light in vegetative mycelia and in aerial tissue of Neurospora crassa wild-type and Δ*ve-1* strains. Download Data Set S2, XLSX file, 3.7 MB. Copyright © 2022 Cea-Sánchez et al. 2022 Cea-Sánchez et al. https://creativecommons.org/licenses/by/4.0/ This content is distributed under the terms of the Creative Commons Attribution 4.0 International license.

The transcriptomic response during conidiation was larger than that in response to light in vegetative mycelia, not only in the number of DEGs but also in fold change levels (Fig. S3 and Data Set S3). During conidiation in the dark, 922 DEGs were identified in the wild type and 1,170 DEGs in the *ve-1* mutant. The conidiation transcriptomes of both strains in the dark largely overlapped (589 genes, about 64% of the wild-type transcriptome). However, 333 genes regulated by conidiation in the dark in the wild-type strain lost this regulation in the Δ*ve-1* mutant, and the Δ*ve-1* mutation led to a new set of 581 genes regulated by conidiation not detected previously in the transcriptome of the wild type. After 30 min of light exposure, the numbers of DEGs increased to 1,038 in the wild type and to 1,557 in the *ve-1* mutant (Fig. S3A and Data Set S3). The differences in the conidiation transcriptomes between the wild type and the Δ*ve-1* mutant support a role for VE-1 in the regulation of transcription during conidiation.

10.1128/mbio.01505-22.8 DATA SET S3 Characterization of the regulation of transcription by conidiation in the Neurospora crassa wild-type and Δ*ve-1* strains. Download Data Set S3, XLSX file, 2.9 MB. Copyright © 2022 Cea-Sánchez et al. 2022 Cea-Sánchez et al. https://creativecommons.org/licenses/by/4.0/ This content is distributed under the terms of the Creative Commons Attribution 4.0 International license.

We identified 77 genes for transcription factors with differential expression during conidiation either in the wild-type or the Δ*ve-1* mutant strain in the dark or the light (Data Set S3). Clustering analysis allowed us to identify TF genes that were highly induced during conidiation in the Δ*ve-1* mutant but only moderately induced in the wild-type strain, such as *fl* and *csp-2*, and TF genes with the opposite expression pattern (induced during conidiation in the wild type but not in the Δ*ve-1* mutant) (Fig. S3C, groups 3 and 4, respectively). In addition, we identified two transcription factor genes, *vib-1* and *sgr-28* (Fig. S3C, group 2), with reduced mRNA accumulation in the Δ*ve-1* mutant in both mycelia and aerial hyphae. Since mutants with mutations in several of these TF genes have alterations in the growth of aerial tissue and the production of conidia (31), they are candidate TFs that may collaborate with VE-1 in the transcriptional regulation of conidiation.

A comparison of the genes regulated during conidiation in the two strains in the dark and the light allowed the identification of a set of 477 DEGs present in all strains and conditions (358 genes upregulated and 119 genes downregulated) (Fig. S3A). This set of conidiation genes did not require the velvet complex for developmental regulation. However, the induction levels for this set of genes were higher in the Δ*ve-1* mutant (median log_2_FCs of 5.86 and 5.79 in the light and the dark, respectively) than in the wild type (median log_2_FCs of 4.70 and 4.52 in the light and the dark, respectively). Repression was also stronger for downregulated genes in the Δ*ve-1* mutant (median log_2_FCs of −4.01 and −3.72 for the Δ*ve-1* mutant versus −3.25 and −2.46 for the wild type in the light and in the dark, respectively) (Fig. S3B). This is consistent with the higher conidiation levels of the Δ*ve-1* mutant.

Our results suggest that VE-1 plays a major role in regulating transcription during conidiation, but not during mycelial growth, by changing the set of regulated genes, including TF genes, and the relative levels of transcription for conidiation-specific genes.

### VE-1 regulates the transcription of methyltransferase genes, including *lae-1*.

**FIG 4:**
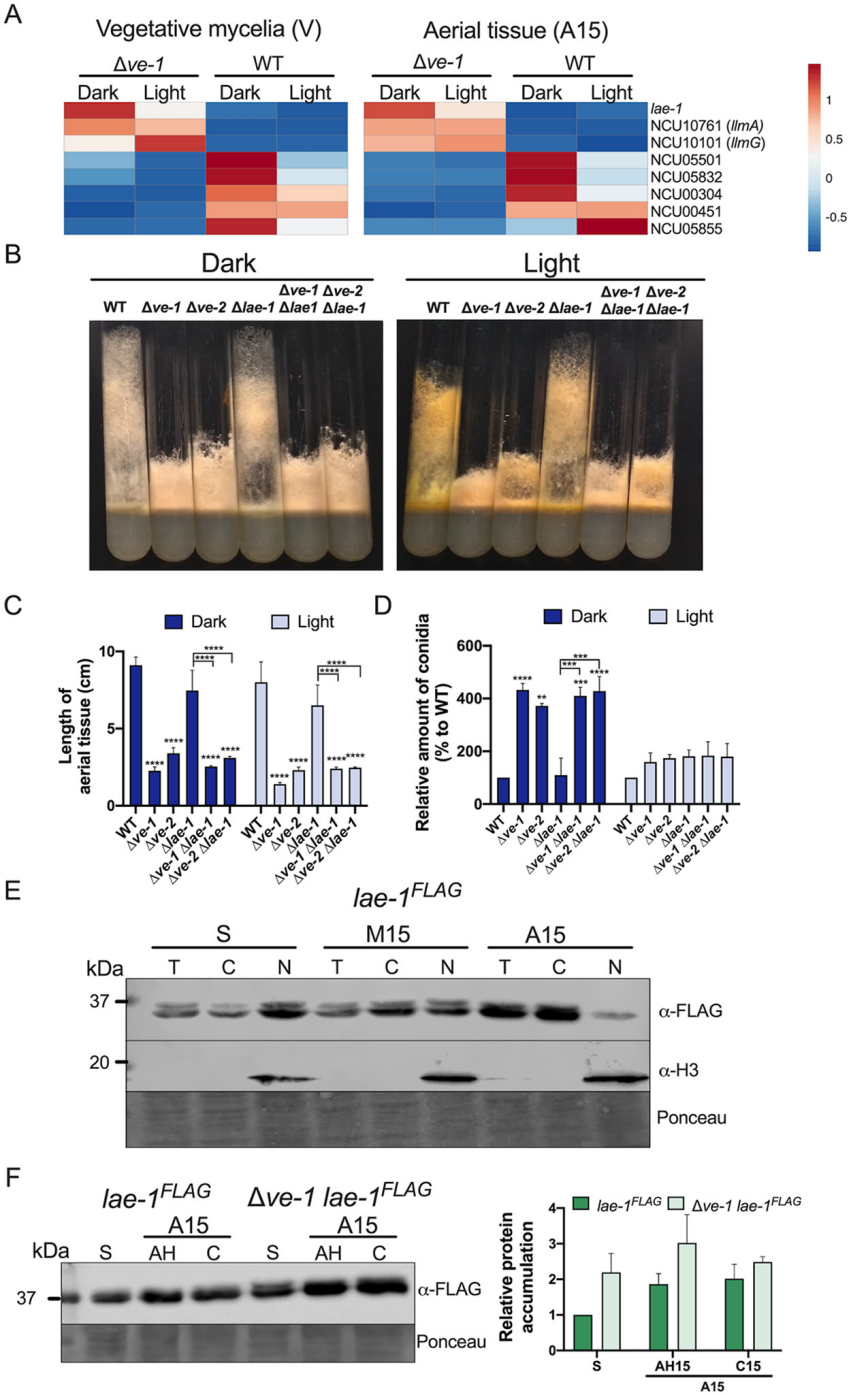
VE-1 regulates transcription of methyltransferase genes. (A) Heat map of relative transcript levels of DEGs encoding methyltransferases in submerged vegetative mycelia (V) or in aerial tissue at 15 h (A15) after induction of conidiation in wild-type and Δ*ve-1* mutant strains. (B) Photographs of culture tube phenotypes of the wild type and Δ*ve-1*, Δ*ve-2*, Δ*lae-1*, and double deletion mutants after 3 days of growth at 30°C in the dark or the light. (C) Lengths of aerial tissue of the wild-type and mutant strains after 3 days of growth at 30°C in the dark or the light. (D) Relative amounts of conidia produced in the wild type and single and double deletion mutants. Conidia from cultures grown in the dark and the light at 30°C were collected after 3 days of incubation, and the amounts plotted relative to the amount produced by the wild type in the dark or the light. (E) Subcellular localization of LAE-1 during conidiation. We collected samples from submerged vegetative mycelia (S), supporting vegetative mycelia (M15), and aerial tissue (A15) from the *lae-1^FLAG^* strain growing in the light. Total protein samples (T) or samples enriched in cytoplasmic (C) or nuclear (N) proteins were separated by SDS-PAGE and hybridized with antibodies specific for FLAG or histone H3. An amount of 70 μg of protein was loaded per lane. As a loading control, we used Ponceau staining of each protein sample. (F) Accumulation of LAE-1 during conidiation. Protein samples from cultures kept in the light were obtained from submerged vegetative mycelia (S). Aerial tissue (A15) was collected after 15 h of induction of conidiation, separated by filtration, and cleaned by centrifugation to obtain separate samples of aerial hyphae (AH) and conidia (C). We used the *lae-1^FLAG^* and Δ*ve-1 lae-1^FLAG^* strains. Proteins were separated by SDS-PAGE and hybridized with an antibody specific for FLAG. An amount of 70 μg of protein was loaded per lane. As a loading control, we used Ponceau staining of each protein sample. Plots show the mean values and standard errors from three independent experiments. Two-way ANOVA comparisons were performed between the values for all strains. Statistical significance is shown only for comparisons between the wild-type and single mutant strains and between every combination of a single mutant with the corresponding double mutant strains (Δ*lae-1* versus Δ*ve-1* Δ*lae-1*, Δ*lae-1* versus Δ*ve-2* Δ*lae-1*, etc.). Significant differences are indicated by asterisks, as follows: *, *P* < 0.05; **, *P* < 0.01; ***, *P* < 0.001; ****, *P* < 0.0001.

During our transcriptomic comparisons between the wild type and the Δ*ve-1* mutant, genes encoding eight methyltransferases, including *lae-1*, showed differential expression levels. While the genome of A. nidulans encodes more than 10 LaeA-like methyltransferases (*llmA* to *llmJ*), some of which are involved in the regulation of development and secondary metabolism (32), 30 homologs of *laeA*-like genes are present in the genome of N. crassa. LAE-1 (NCU00646) is a 326-aa protein with 42% identity with A. nidulans LaeA, and deletion of *lae-1* results in defects in asexual development and a moderate decrease in the amount of conidia (28). We observed that *lae-1* was upregulated in vegetative mycelia of the Δ*ve-1* mutant, together with the genes for two other LaeA-like methyltransferases from A. nidulans, NCU10101 (*llmG*) and NCU10761 (*llmA*) (Fig. 4A). The opposite effect was observed for the homologs of five other A. nidulans methyltransferases: the genes for two LlmB-like methyltransferases (NCU05501 and NCU05832), the *O*-methyltransferase type (NCU05855), and two *laeA*-like genes (NCU00541 and NCU00304) were downregulated in the Δ*ve-1* mutant. In addition, the methyltransferases NCU10101 and NCU10761 were upregulated during conidiation in the Δ*ve-1* mutant (Fig. 4A). Our results suggested that VE-1 may have a regulatory effect on the transcription of several methyltransferase genes, including *lae-1*.

Given the role of LAE-1 as part of the velvet complex (28) and the possible regulatory relationship with VE-1, we characterized the developmental phenotypes of a Δ*lae-1* mutant in combination with mutations in *ve-1* or *ve-2*. The developmental phenotypes of mutations in either Δ*ve-1* or Δ*ve-2* (shorter aerial tissue and increased conidiation in the dark) were not suppressed by a deletion of *lae-1*. The two double mutants (Δ*ve-1* Δ*lae-1* and Δ*ve-2* Δ*lae-1*) showed the same developmental phenotypes as the Δ*ve-1* or Δ*ve-2* single mutants (Fig. 4B and C). The absence of a clear conidiation phenotype in the Δ*lae-1* mutant alone or in combination with mutations in *ve-1* or *ve-2* suggests a major role for VE-1 and VE-2 in the developmental regulatory activity by the velvet complex (Fig. 4D). Using a FLAG-tagged *lae-1* strain, we observed accumulation of LAE-1 during asexual development that followed a similar pattern to that of VE-2. The levels of LAE-1 in both vegetative mycelia (submerged and supporting mycelia) and aerial tissue were similar, with small increases in the amounts of LAE-1 in aerial hyphae and conidia 15 and 24 h after the induction of asexual development; LAE-1 localized in the cytoplasmic and nuclear fractions, but it was less abundant in nuclei of aerial tissue (Fig. 4E and F and Fig. S2). Developmental regulation of *lae-1* was not observed by transcriptome analysis (Data Set S3), but the increases in *lae-1* levels in the Δ*ve-1* strain suggested that the absence of VE-1 altered the accumulation of LAE-1. Indeed, we observed a 2-fold increase in the LAE-1 protein level in the absence of VE-1 in submerged vegetative mycelia and a slight increase in aerial tissue in the Δ*ve-1* mutant (Fig. 4F). Our results suggest that VE-1 plays a regulatory role not only in the transcription of *lae-1* but also in the accumulation of LAE-1.

### Role of VE-1 in aerial tissue length: identification of VIB-1.

As shown by the experiments described above, the deletion of *ve-1* produced two major phenotypes during growth on solid medium: a drastic reduction in the length of aerial tissue and increased conidiation in the dark. It is possible that some of the genes misregulated in the Δ*ve-1* mutant could play a role in the developmental alterations produced by a nonfunctional velvet complex. A number of genes that were identified as DEGs in our transcriptomic results were selected based on the following criteria: (i) genes up- and downregulated by conidiation exclusively in the wild type with differences in log_2_FCs of >3 and (ii) genes showing differences in mRNA accumulation (up and down) in aerial tissue in the dark and the light with log_2_FCs of >3 after pairwise comparisons between the wild type and the Δ*ve-1* mutant. We then used the collection of N. crassa deletion mutants (33) with the most promising candidate genes to characterize their developmental phenotypes with the aim of identifying mutants with a phenotype similar to that of the Δ*ve-1* mutant.

**FIG 5:**
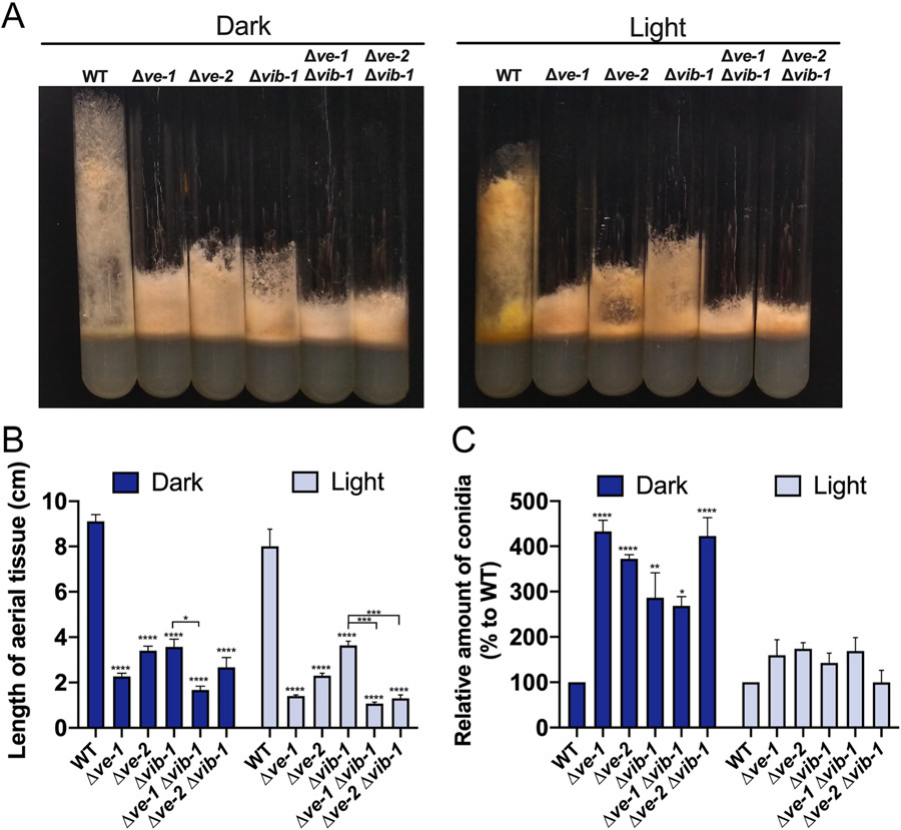
VE-1 regulates *vib-1* during the development of aerial tissue and conidiation. (A) Photographs of culture tube phenotypes of the wild type and Δ*ve-1*, Δ*ve-2*, Δ*vib-1*, and double deletion mutants after 3 days of growth at 30°C in the dark or the light. (B) Lengths of aerial tissue of wild-type and mutant strains after 3 days of growth at 30°C in the dark or the light. (C) Relative amounts of conidia produced in the wild type and single and double deletion mutants. Conidia from cultures grown in the dark and the light at 30°C were collected after 3 days of incubation, and the amounts plotted relative to the amount produced by the wild type in the dark or the light. Plots show the mean values and standard errors from three independent experiments. Two-way ANOVA comparisons were performed between the values for all strains. Statistical significance is shown only for comparisons between the wild-type and single mutant strains and between every combination of a single mutant with the corresponding double mutant strains (Δ*vib-1* versus Δ*ve-1* Δ*vib-1*, Δ*vib-1* versus Δ*ve-2* Δ*vib-1*, etc.). Significant differences are indicated by asterisks, as follows: *, *P* < 0.05; **, *P* < 0.01; ***, *P* < 0.001; ****, *P* < 0.0001.

We identified 268 genes whose deletion strains were available in the collection of N. crassa deletion mutants (Data Set S4); the phenotype of each strain was assayed by growth in slants and in 55-mm petri dishes. Mutants with defects in the length of aerial tissue and in the pattern of conidiation across the petri dish were further selected for phenotypic confirmation. Eight mutants showed alterations in their growth pattern, of which three strains displayed a growth phenotype similar to that of the Δ*ve-1* strain. One of these three mutants had a growth phenotype that did not segregate with the hygromycin resistance marker used for deleting the gene, while the gene deleted in the second mutant was expressed weakly under all conditions. The third candidate gene was NCU03725, which had lower expression in the Δ*ve-1* mutant than in the wild type during conidiation (log_2_FCs of −2.37 in the dark −3.20 in the light) (Data Set S1). Mutation of this gene resulted in a defect in the length of aerial tissue and a conidiation pattern intermediate between those of the Δ*ve-1* mutant and the wild-type strain (Fig. 5). The gene NCU03725 has been characterized previously as *vib-1* (vegetative incompatibility blocked-1) and encodes a homolog of Saccharomyces cerevisiae NDT80, a transcription factor involved in the regulation of genes during meiosis (34). VIB-1 does not play a role in meiosis in N. crassa (35) but has been identified as a major regulator of responses to nitrogen and carbon starvation (36) and as essential for the expression of genes involved in non-self-recognition and cell death in N. crassa (37, 38). In addition, VIB-1 has been described as a negative regulator of conidiation (39). We hypothesize that VE-1 could regulate conidiation by acting as a positive regulator of the expression of *vib-1*. To test this hypothesis, we created double mutants with mutations of *ve-1* and *ve-2* along with the mutation of *vib-1*. All single and double (Δ*ve-1* Δ*vib-1* and Δ*ve-2* Δ*vib-1*) mutants showed reduced length of aerial tissue both in the light and the dark and increased conidiation in the dark, like each of the single Δ*ve-1* and Δ*ve-2* mutants (Fig. 5). The two double mutants (Δ*ve-1* Δ*vib-1* and Δ*ve-2* Δ*vib-1* strains) both showed a decrease in the length of aerial tissue compared to that of the Δ*vib-1* mutant alone (with the exception of the Δ*ve-2* Δ*vib-1* mutant in the dark), suggesting that VIB-1 may operate, at least partially, in a different pathway for the regulation of asexual development and that control of aerial hyphal growth requires both VE-1 and VIB-1 in N. crassa. Previously, Wu et al. identified 1,742 VIB-1 binding sites by DNA affinity purification sequencing (DAP-seq) (36); VIB-1 bound the promoters of 94 (dark) and 86 (light) DEGs out of 237 and 343 genes that required VE-1 for proper expression during conidiation in the dark and the light, respectively (Fig. 3C). This accounted for 39% (dark) and 25% (light) of the DEGs, which supports the proposal that a significant proportion of the VE-1 regulon requires VIB-1.

10.1128/mbio.01505-22.9 DATA SET S4 List of N. crassa mutants screened for *ve-1* phenotype. Download Data Set S4, XLSX file, 0.04 MB. Copyright © 2022 Cea-Sánchez et al. 2022 Cea-Sánchez et al. https://creativecommons.org/licenses/by/4.0/ This content is distributed under the terms of the Creative Commons Attribution 4.0 International license.

### Role of VE-1 in conidiation: regulation of *fl* and microconidiation.

**FIG 6:**
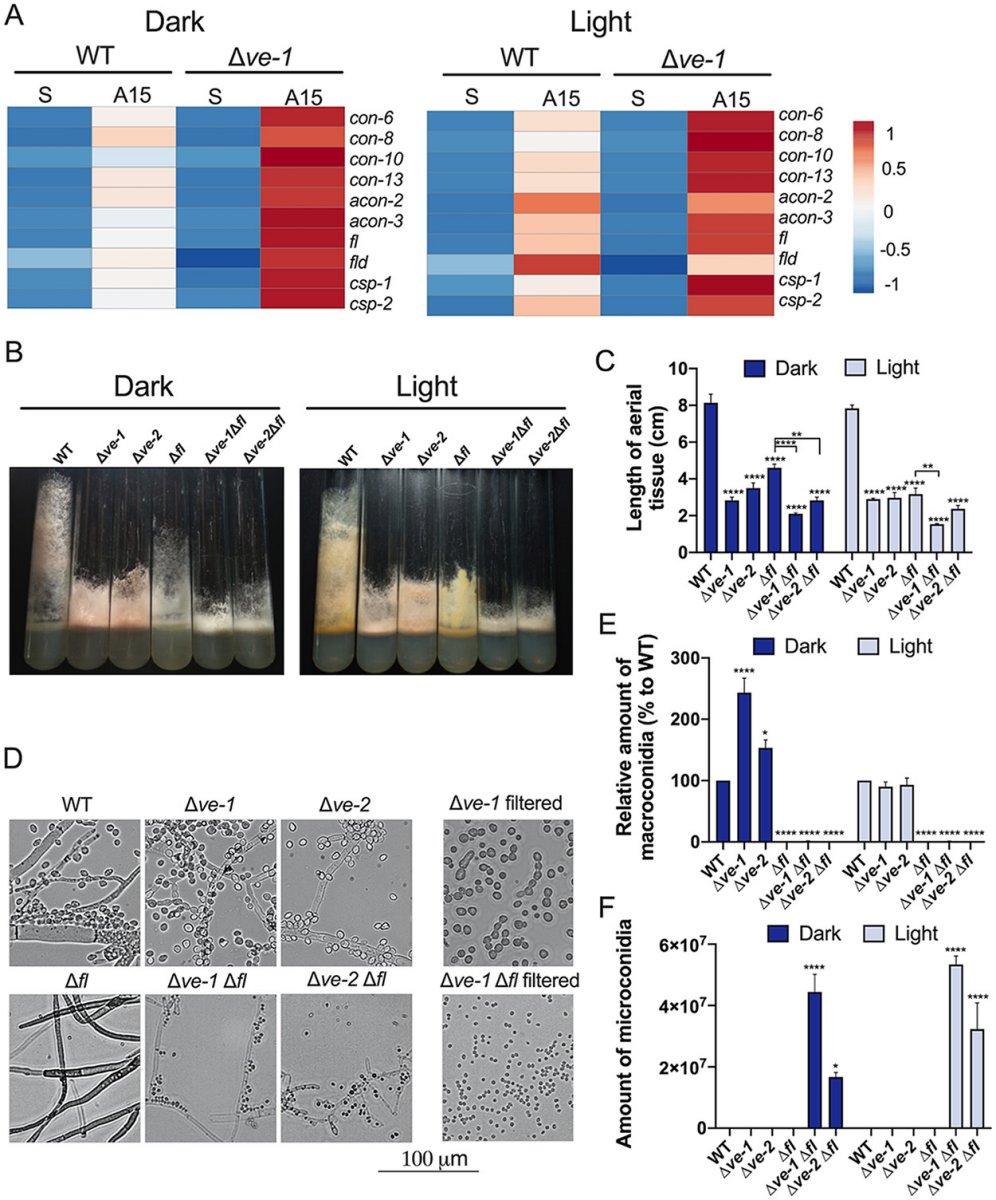
The velvet complex regulates microconidiation. (A) Heat map of relative transcript levels of conidiation-related genes in submerged vegetative mycelia (S) or in aerial tissue 15 h (A15) after induction of conidiation in the dark or after exposure to light for 30 min prior to collection. We used the wild-type and the Δ*ve-1* mutant strains. (B) Photographs of culture tube phenotypes of the wild type and Δ*ve-1*, Δ*ve-2*, Δ*fl*, and double deletion mutants after 3 days of growth at 30°C in the dark or the light. (C) Lengths of aerial tissue of the wild-type and mutant strains after 3 days of growth at 30°C in the dark or with light. (D) Vegetative hyphae and conidiation (macro- and microconidiation) in samples of each single and double mutant. Macro- and microconidia from Δ*ve-1* and Δ*ve-1* Δ*fl* strains were obtained after filtration with cheese cloth. (E) Relative amounts of macroconidia in the wild type and single and double deletion mutants. Results are plotted relative to the amount produced by the wild type in the dark or the light. (F) Amounts of microconidia in the wild type and single and double deletion mutants. Conidia (macro- and microconidia) were collected from cultures grown in the dark and the light at 30°C after 3 days of incubation. Plots show the mean values and standard errors from three independent experiments. Two-way ANOVA: *, *P* < 0.05; **, *P* < 0.01; ***, *P* < 0.001; ****, *P* < 0.0001.

Genetic analysis has led to the proposal that FL and VIB-1 participate in the same pathway for the regulation of conidiation and that FL partially activates conidiation by repressing VIB-1 (39). We have shown that VE-1 is required for the transcription of *vib-1* and that VE-1 is a repressor of *fl* and other conidiation genes (Fig. 6A; Fig. S3C and Data Set S3). FL is a key transcription factor in the regulation of conidiation since *fl* mutants are blocked in the transition from minor to major constrictions during the development of conidia (12, 14). To characterize a possible interaction between FL and VE-1 in the regulation of conidiation, we created double mutant strains with mutations in *ve-1* or *ve-2* along with *fl*.

The Δ*ve-1* Δ*fl* and Δ*ve-2* Δ*fl* double mutant strains displayed the short aerial phenotype that is characteristic of each of the Δ*ve-1*, Δ*ve-2*, and Δ*fl* single mutants (Fig. 6B and C), but the double mutants showed shorter aerial tissue than the Δ*fl* strain (with the exception of the Δ*ve-2* Δ*fl* mutant in the light), suggesting that VE-1, VE-2, and FL act, at least partially, in different pathways to regulate the growth of aerial tissue. The Δ*ve-1* Δ*fl* and Δ*ve-2* Δ*fl* strains lacked macroconidial production, like the Δ*fl* single mutant, but the double mutants produced large amounts of microconidia, unlike each single mutant (Fig. 6D to F). The increase in microconidiation in the Δ*ve-1* Δ*fl* and Δ*ve-2* Δ*fl* mutants suggests that the velvet complex acts as a repressor of microconidiation when the macroconidiation pathway is blocked by a deletion of *fl*. Our results support the proposal that the velvet complex plays a key role in the transcriptional regulation of conidiation and the balance between macro- and microconidiation in N. crassa.

## DISCUSSION

Velvet proteins and the velvet complexes regulate development, secondary metabolism, and pathogenicity in fungi, but the specific roles of the velvet proteins may differ from one species to another, which is particularly evident in the regulation of fungal development. For example, in A. nidulans, VeA plays a crucial role in conidiation in response to red light (40), and its role depends on its subcellular localization, which is regulated by light (18, 21). The VeA homolog is required for conidiation in Penicillium chrysogenum (41) and Trichoderma reesei (42). However, deletion of the *veA* homolog results in a hyperconidiating phenotype in Aspergillus fumigatus (43). In several species of Fusarium, mutations in *veA* homologs result in alterations in conidiation that are specific for each species. For example, deletion of the *veA* homolog in Fusarium verticillioides and Fusarium fujikoroi resulted in increased macroconidiation and decreased microconidiation (44, 45), but the same deletion in Fusarium oxysporum resulted in reductions in aerial hyphal formation and conidial production (46). VeA and homologous proteins act as global regulators of fungal development (16, 17). Here, we show that in N. crassa, a mutation in *ve-1* or *ve-2* results in a reduced length of aerial tissue and increased conidiation in the dark by modulating the transcriptional responses.

The N. crassa velvet complex is composed of VE-1, VE-2, and LAE-1 (28). In addition, the velvet protein VOS-1 interacts with VE-1 and VE-2 (28). Our observation that mutations in *ve-1* or *ve-2* resulted in alterations of asexual development, while mutants with mutations in *vos-1* or *lae-1* had a wild-type phenotype, suggests a role in the regulation of asexual development for a regulatory complex composed of VE-1 and VE-2. The stability of the levels of VE-2 during conidiation, unlike the levels of VE-1, suggests that VE-1 acts as the limiting component in the formation of complexes with VE-2 and LAE-1 and that the activity of these complexes is regulated by the availability of VE-1.

Given the key role that VE-1 should have in the formation and activity of the velvet complex, we characterized the transcriptome of a Δ*ve-1* mutant strain during vegetative growth and in the early stages of conidiation. Our results show that the absence of VE-1 led to major changes in the conidiation transcriptome, suggesting that the developmental phenotypes we observed are due to changes in transcription. Given the DNA-binding capability of the velvet domain (19), it is conceivable that VE-1 regulates transcription directly by binding to target promoters, as has been shown for VeA in A. nidulans (23). VE-1 may also indirectly regulate conidiation-dependent transcription factors, as we identified a set of transcription factor genes with altered transcriptional profiles in the absence of VE-1 that could participate in the regulation of conidiation in coordination with VE-1. In addition, the absence of VE-1 led to changes in the accumulation of mRNAs for the LAE-1 gene (and genes encoding other methyltransferases) and the VOS-1 gene, suggesting that VE-1 also regulates the composition of its interacting partners through transcriptional regulation.

We have identified the transcription factor VIB-1 as a candidate protein that participates with VE-1 in the regulation of development in N. crassa. *vib-1* was initially identified in N. crassa as a gene required for the regulation of vegetative incompatibility and encodes a transcription factor (37). More recently, it was shown that VIB-1 directly binds and regulates genes associated with nutrient assimilation and regulation of genes associated with plant cell wall deconstruction (36). In particular, two genes encoding LaeA-like methyltransferase domains (NCU05832 and NCU05501) that were downregulated in the Δ*ve-1* mutant are in the core VIB-1 regulon, while four additional LaeA-like genes (NCU04909, NCU04717, NCU04707, and NCU01148) are direct targets of VIB-1. Genetic interactions between *vib-1* and *fl*, a key regulator of conidiation that encodes a conidiation-dependent transcription factor, led to the proposal that FL acts as a negative regulator of VIB-1 and that VIB-1 is a repressor of conidiation (39). In this hypothesis, the aconidial phenotype of *fl* is due to an excessive repression of conidiation by VIB-1. Our transcriptomic analysis shows that *vib-1* is downregulated in the absence of VE-1, while *fl* and other conidiation genes are upregulated, suggesting that VE-1 participates in the regulation of conidiation by repressing the transcription of *fl*, among other TF genes, and activating the transcription of *vib-1*.

When the *fl* mutation in the aconidial Δ*fl* strain was combined with the deletion of either *ve-1* or *ve-2*, the resulting double mutant strains produced large amounts of conidia. However, instead of producing large multinucleate macroconidia, the double Δ*ve-1* Δ*fl* and Δ*ve-2* Δ*fl* strains produced large amounts of microconidia. These are small and uninucleate (unlike macroconidia) and are only observed under specific environmental conditions with high humidity, low temperature, and limiting nutrients (9). Under our growth conditions, we did not detect microconidia in the wild-type or the *fl* mutant strain. In addition, we could not detect microconidia in the single Δ*ve-1* or Δ*ve-2* strains, suggesting that the absence of VE-1 or VE-2 alone was not sufficient to induce microconidiation. Instead, the suppression of macroconidiation by the absence of FL and of VE-1 or VE-2 led to a misregulation of microconidiation and an abundant production of microconidia in these double mutant strains.

How could VE-1 and VE-2 regulate microconidiation? It is possible that the VE-1/VE-2 complex may regulate microconidiation by controlling the transcription of key regulatory genes, as observed in the regulation of macroconidiation. Two strains that overproduce microconidia have been reported in the past, and they carry mutations in *fl* together with mutations in other genes (*pe;fl* and *dn;fl*) (9). The presence of a mutation in *fl* in several microconidiating strains suggests that in order to overproduce microconidia, N. crassa requires a blockage of macroconidiation and mutations in other regulatory genes. Genetic mapping and mutant sequencing identified *pe* as an allele of *ve-1* (S. Baker, D. Wright, and M. Freitag, personal communication), and it is possible that *dn* is an allele of *ve-2* or a novel gene involved in the regulation of microconidiation. In addition, several conidiation (*con*) genes are expressed during macro- and microconidiation (11), suggesting common regulatory elements, such as VE-1 and VE-2. It is tempting to hypothesize that the VE-1/VE-2 complex participates in the repression of microconidiation by regulating, in addition, the transcription of microconidiation-specific regulators yet to be identified.

## MATERIALS AND METHODS

### Strains and culture conditions.

Fungal strains used in this work are listed in Table S1. Escherichia coli DH5a was used for plasmid manipulations. S. cerevisiae strain FY834 (*MAT*α *his3Δ200 ura3-52 leu2Δ1 lys2Δ202 trp1Δ63*) was employed for assembling PCR fragments for gene tagging in N. crassa. Strain manipulation and growth medium preparation followed standard procedures and protocols (47). General procedures and media for N. crassa are available at the Fungal Genetic Stock Center website (www.fgsc.net/Neurospora/NeurosporaProtocolGuide.htm).

10.1128/mbio.01505-22.4 TABLE S1 List of strains used in this study. Download Table S1, PDF file, 0.1 MB. Copyright © 2022 Cea-Sánchez et al. 2022 Cea-Sánchez et al. https://creativecommons.org/licenses/by/4.0/ This content is distributed under the terms of the Creative Commons Attribution 4.0 International license.

For the light induction experiments in vegetative mycelia, 10^6^ conidia were inoculated into 25 mL of Vogel’s liquid medium in 90-mm plates, which were incubated in complete darkness at 22°C for 48 h. The cultures were then exposed to the light for 30 min. A control treatment was always kept in the dark. Vegetative mycelia were collected, dried on paper, frozen in liquid nitrogen, and stored at −80°C.

For the induction of conidiation, we modified the method of Bailey and Ebbole (14). A total of 10^7^ conidia were inoculated into 200 mL of Vogel’s liquid medium in 500-mL flasks and incubated for 24 h at 30°C with agitation. Vegetative mycelia were collected by filtration onto filter paper, covered with an additional layer of filter paper, and placed on top of 25 mL of Vogel’s solid medium in 90-mm plates to induce conidiation. Cultures were incubated at 30°C for 15 h or 24 h to allow the aerial hyphae to develop and grow through the filter paper. Samples of the aerial tissue (aerial hyphae and developing conidia) located on top of the filter papers and the supporting vegetative mycelial mat located between the two filter papers were collected separately. In some experiments, conidia were separated from aerial hyphae in the aerial tissue by filtration through cheesecloth and purified by centrifugation. Samples of submerged vegetative mycelia from the initial liquid cultures were collected as vegetative controls. Cultures were either kept in the dark or under constant light or kept in the dark and then exposed to 30 min of light prior to collection. Samples were dried on paper, frozen in liquid nitrogen, and stored at −80°C.

Constant illumination was provided by a set of fluorescent lamps (containing 1 W/m^2^ of blue light). All the manipulations in the dark were performed under red light. At least three independent biological replicates were employed in RNA or protein quantification experiments.

### Plasmid construction and gene replacement.

The generation of an N. crassa strain with genes tagged by the FLAG epitope after replacement of the wild-type allele was performed by following a previously described method (48) with minor modifications. The generation of knock-in tagging cassettes involved the generation of three DNA segments of about 1.2 kb in length, including one segment corresponding to DNA upstream from the stop codon (5′ untranslated region [5′-UTR] of *ve-1*), a segment corresponding to the FLAG epitope and hygromycin resistance gene fusion (10×Gly::3×FLAG::*hph*), and a final segment corresponding to DNA downstream from the stop codon (3′-UTR of gene of interest). Each flank was obtained by PCR using primers (Table S2) with a tail complementary to the tagging fragment obtained by digestion of plasmid DNA (plasmid p3xFLAG::hph::loxP; GenBank accession number FJ457009). The three fragments (two PCR products and a linear digested fragment) and the linearized plasmid pRS426 were fused to make the knock-in cassettes by recombination in an auxotrophic strain of S. cerevisiae (49). The FLAG epitope was fused to the carboxy end of the protein of interest by a polyglycine adaptor, followed by the hygromycin resistance gene (*hph*) for the selection of transformed N. crassa strains. Transformants were backcrossed with the wild-type strain to isolate homokaryotic strains. Genetic crosses were performed to obtain strains that carried the desired new allele with the coding sequence for the FLAG epitope along with other selected mutations. All strains were verified by PCR and Western blotting.

10.1128/mbio.01505-22.5 TABLE S2 List of primers used in this study. Download Table S2, PDF file, 0.1 MB. Copyright © 2022 Cea-Sánchez et al. 2022 Cea-Sánchez et al. https://creativecommons.org/licenses/by/4.0/ This content is distributed under the terms of the Creative Commons Attribution 4.0 International license.

### RNA isolation and quantitative RT-PCR.

Mycelia were disrupted by two 0.5-min pulses in a cell homogenizer (FastPrep-24; MP Biomedicals) with 1.5 g of zirconium beads (0.5-mm diameter) in 1.9-mL screw-cap tubes by using the RNeasy plant minikit (Qiagen) with the RLC buffer following the manufacturer’s procedure. The extracts in screw-cap tubes were clarified by centrifugation (13,000 rpm) for 5 min prior to RNA purification. The RNA samples were treated with DNase I (USB) prior to use in reverse transcription (RT)-PCR experiments. The quality of the RNA was confirmed by inspecting the absorption spectra at 260 nm as recommended by the supplier of the RT-PCR kit and equipment. The primers employed for quantitative RT-PCR are detailed in Table S2. Quantitative RT-PCR experiments were performed using one-step RT-PCR in a LightCycler 480 II instrument (Roche) by using the one step SYBR PrimeScript RT-PCR kit (TaKaRa Bio, Inc.), 0.2 μM each primer, and 12.5 ng of RNA in a 10-μL reaction mixture volume. The reaction settings consisted of 5 min at 42°C, followed by 10 s at 95°C, and then 40 cycles of DNA amplification (5 s at 95°C and 20 s at 60°C). After each PCR, we performed a melting curve analysis to show the specific amplification of single DNA segments and the absence of nonspecific amplified DNA. The fluorescence signal obtained for each gene was normalized to the corresponding fluorescence signal obtained with *tub-2* to correct for sampling errors. Expression data are the average values from at least three independent experiments.

### Protein isolation and detection.

Proteins were extracted from mycelia by previously described methods (50) using a modified lysis buffer (50 mM HEPES, pH 7.4, 137 mM NaCl, 10% glycerol, 5 mM EDTA, 29.3 μM phenylmethyl-sulfonyl fluoride [PMSF], 6.3 μM leupeptin, 4.4 μM pepstatin A). Total proteins were subjected to SDS-PAGE on 10% (29:1) acrylamide-bisacrylamide gels and transferred to nitrocellulose membranes. Equal loading was confirmed by staining the hybridization membrane with Ponceau S solution. Membranes were hybridized with monoclonal antibody against FLAG (F3165, 1:10,000; Sigma) or polyclonal antibody against histone H3 (AB1791, 1:5,000; Abcam). Horseradish peroxidase (HRP)-conjugated goat anti-mouse IgG (1721011, 1:10,000; Bio-Rad) was used as a secondary antibody for anti-FLAG antibody, and goat anti-rabbit IgG (1706515, 1:10,000; Bio-Rad) was used as the secondary antibody for anti-HA and anti-histone H3 antibody detections.

Nuclear fraction purifications were performed as developed by Baum and Giles (51) with minor modifications (52, 53). Mycelia were pulverized to a fine powder in liquid nitrogen. Eight milliliters of buffer A (1 M sorbitol, 7% [wt/vol] Ficoll, 20% [vol/vol] glycerol, 5 mM magnesium acetate, 5 mM EGTA, 3 mM calcium chloride, 50 mM Tris-HCl, pH 7.5) was added to the powder. Once homogenized, the mixture was filtered through gauze and two volumes of buffer B (10% [vol/vol] glycerol, 5 mM magnesium acetate, 5 mM EGTA, 25 mM Tris-HCl, pH 7.5) was added to the filtrate. The mix of filtrate and buffer B was added to 10.4 mL buffer C (1:1.7 buffers A/B) slowly, to prevent the liquid phases from mixing. This step was performed on 25- by 89-mm centrifuge tubes (Beckman). Centrifugation was performed at 3,000 × *g* at 4°C. A 1-mL sample was collected from the supernatant and designated total cell extract. The remainder was added to 5 mL of 1 M sucrose gradient (1 M sucrose, 10% [vol/vol] glycerol, 5 mM magnesium acetate, 1 mM dithiothreitol [DTT], 25 mM Tris-HCl, pH 7.5) slowly, to prevent mixing of liquid phases. Centrifugation was performed for 30 min at 9,400 × *g* at 4°C. The resulting supernatant was collected and designated the cytoplasmic fraction. The pellet was suspended in 500 μL buffer D (50 mM Tris HCl, pH 7.5, 300 mM NaCl, 1.5 mM MgCl_2_, 0.5% NP-40) and designated the nuclear fraction. Samples of the total cell extract, cytoplasmic fraction, and nuclear fraction were quantified.

### RNA-seq and statistical analysis.

Poly(A) selection and preparation of cDNA libraries for RNA were done according to the Illumina mRNA sample preparation guide. Sequencing was performed on the Illumina Genome Analyzer (Illumina HiSeq version 4) by the Next Generation Sequencing Facility at Vienna BioCenter Core Facilities (VBCF), a member of the Vienna BioCenter (VBC), Austria. Single-end reads of 100 nucleotides each were mapped against the N. crassa OR74A genome (NC12.37) obtained from the JGI MycoCosm web portal https://mycocosm.jgi.doe.gov/Neucr2/Neucr2.home.html (54) by using TopHat 2.11 (55). The read quality was checked using FastQC 0.11.8 (http://www.bioinformatics.babraham.ac.uk/projects/fastqc/), and automatic preprocessing of the reads was carried out with AfterQC 0.9.6 (56). SeqMonk version 1.45.4 (https://www.bioinformatics.babraham.ac.uk/projects/seqmonk/) was used for visualization and quantification of BAM (binary alignment map) files. Transcript features were generated over genes. Quantification was performed using the RNA-seq quantification pipeline on merged transcripts, counting reads over exons and assuming opposing-strand specificity. Differential gene expression analysis was done by using DESeq2 (57). Genes with a log_2_FC of ≥2 or ≤−2 and adjusted *P* values below 0.001 after Benjamin and Hochberg correction were designated differentially expressed genes (DEGs) for comparison between conditions. Three different conditions were analyzed for each strain (Fig. 3A): genes regulated by light during vegetative growth (light regulation experiment) and genes regulated by conidiation (developmental regulation experiment) either in complete darkness for 15 h (A15 dark) or with development in the dark followed by 30 min of light exposure (A15 light). In addition, differential gene expression analysis was carried out to examine pairwise differences between wild-type and Δ*ve-1* strains under different developmental conditions.

Genes showing significant differences in relative expression levels (DEGs) were classified according to their known or putative gene information in the integrated functional genomic database FungiDB (58). All the genes selected were used to create Venn diagrams using http://www.venndiagrams.net/, followed by GO analysis using the FungiFun2 database (59) for enrichment of GO terms into biological processes, cellular components, and molecular functions. The significantly associated gene ontology terms (adjusted *P* value of <0.05) were imported to Revigo (reduce and visualize Gene Ontology), where they were clustered based on their relatedness and any redundancy was removed (60). Heat maps were generated with log_2_ transcripts per kilobase per million (TPM) values using ClustVis (61).

### Conidiation.

For the quantification of conidiation, we prepared 10-mL tubes with 4 mL of Vogel’s solid medium. Each tube was inoculated with 10^4^ conidia and incubated at 30°C in the dark or in constant light. Conidia were isolated after the addition of 5 mL of water and gentle shaking of each tube. Each conidial suspension was collected and filtered through cheesecloth. Each sample was centrifuged and resuspended in sterile water three times prior to quantification in a hemocytometer. We stored conidial samples at 4°C and assayed their viability by plating diluted samples in sorbose agar to induce colonial growth. The plates were incubated at 34°C for 2 days, and the number of N. crassa colonies counted to quantify the number of viable conidia after each period of storage at 4°C.

### Data availability.

RNA-seq results have been deposited in the Gene Expression Omnibus (GEO) database under accession number GSE180332.
