# Supplementary figures and images for: Transcriptional Regulation by the Velvet Protein VE-1 during Asexual Development in the Fungus Neurospora crassa
Source: mBio. 2022 Aug 1;13(4):e01505-22. doi: 10.1128/mbio.01505-22 (PMC9426599; doi:10.1128/mbio.01505-22)

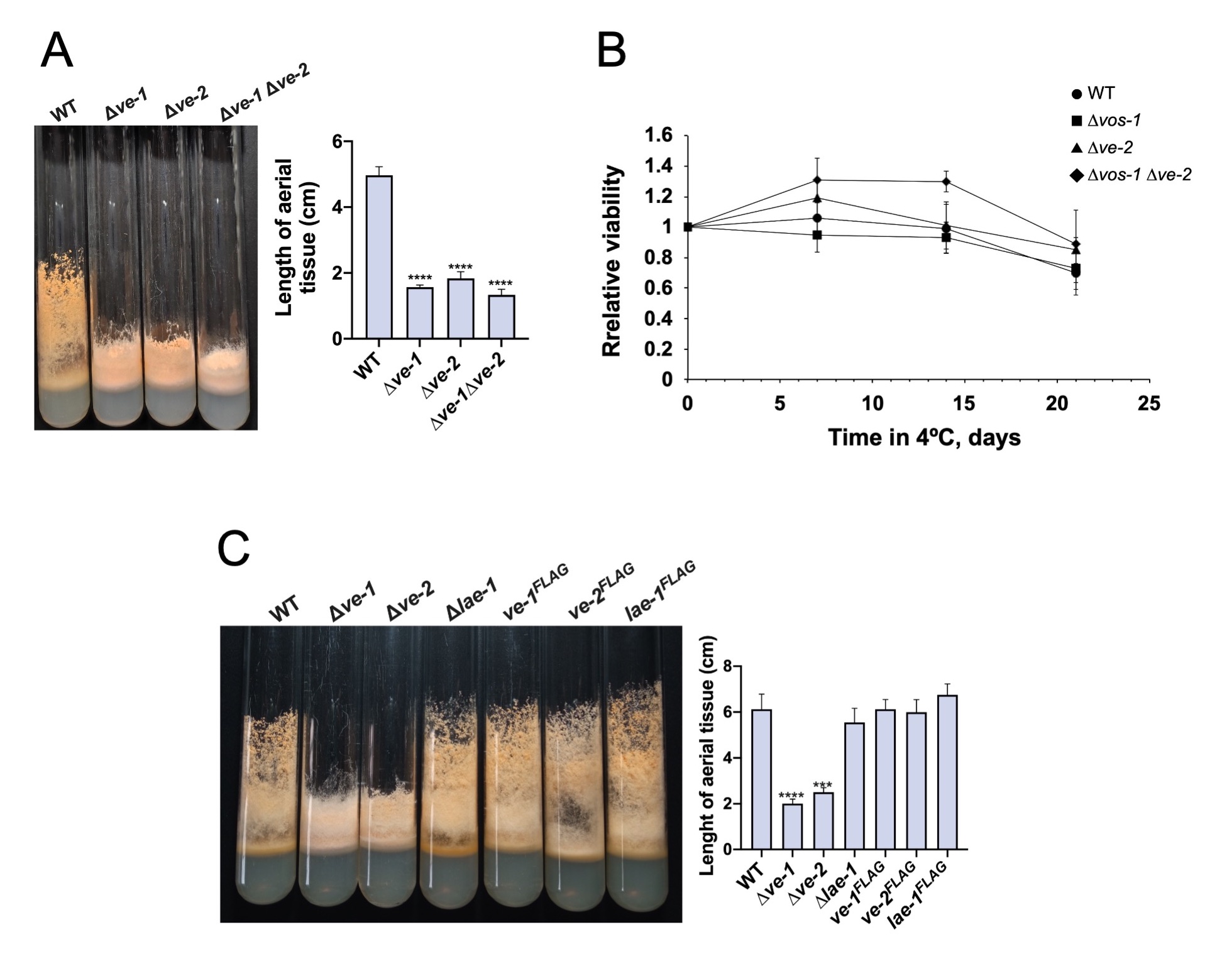

Supplement: FIG S1 [file mbio.01505-22-s0001.jpg]

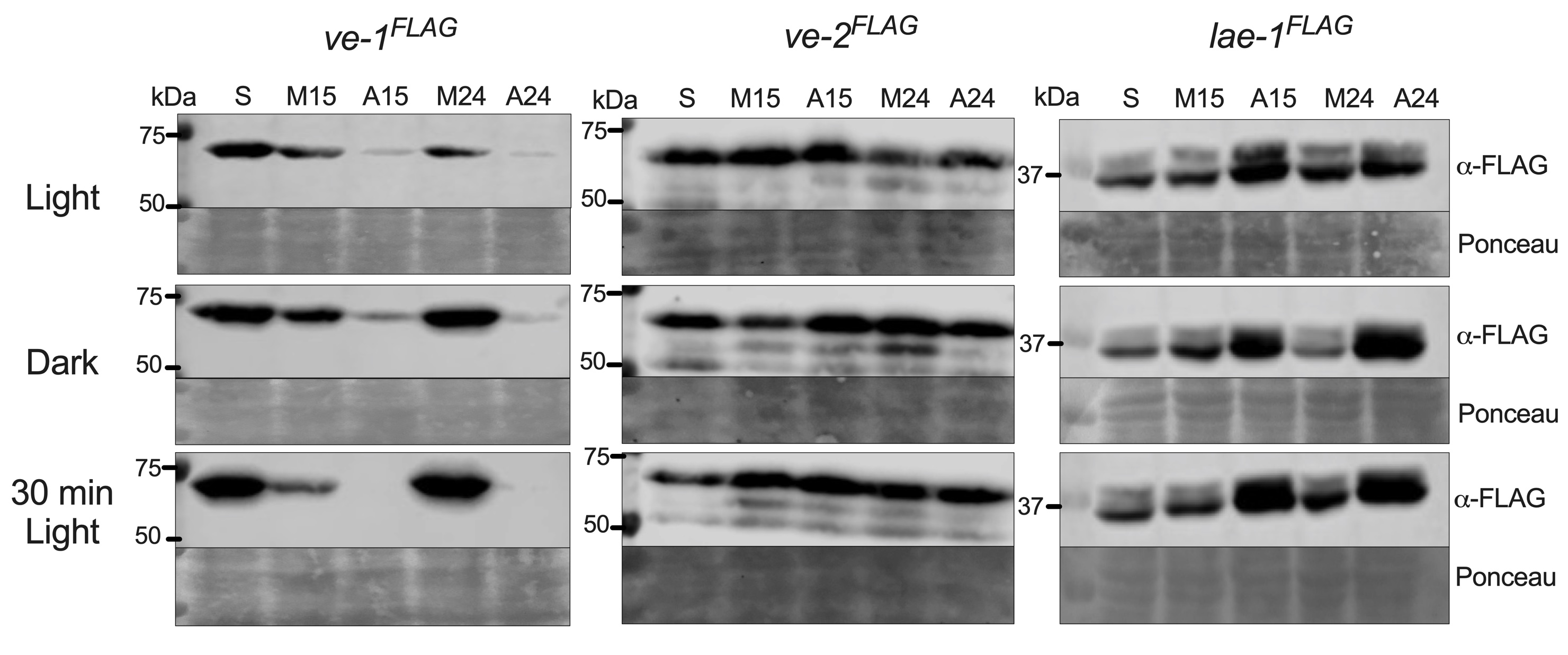

Supplement: FIG S2 [file mbio.01505-22-s0002.jpg]

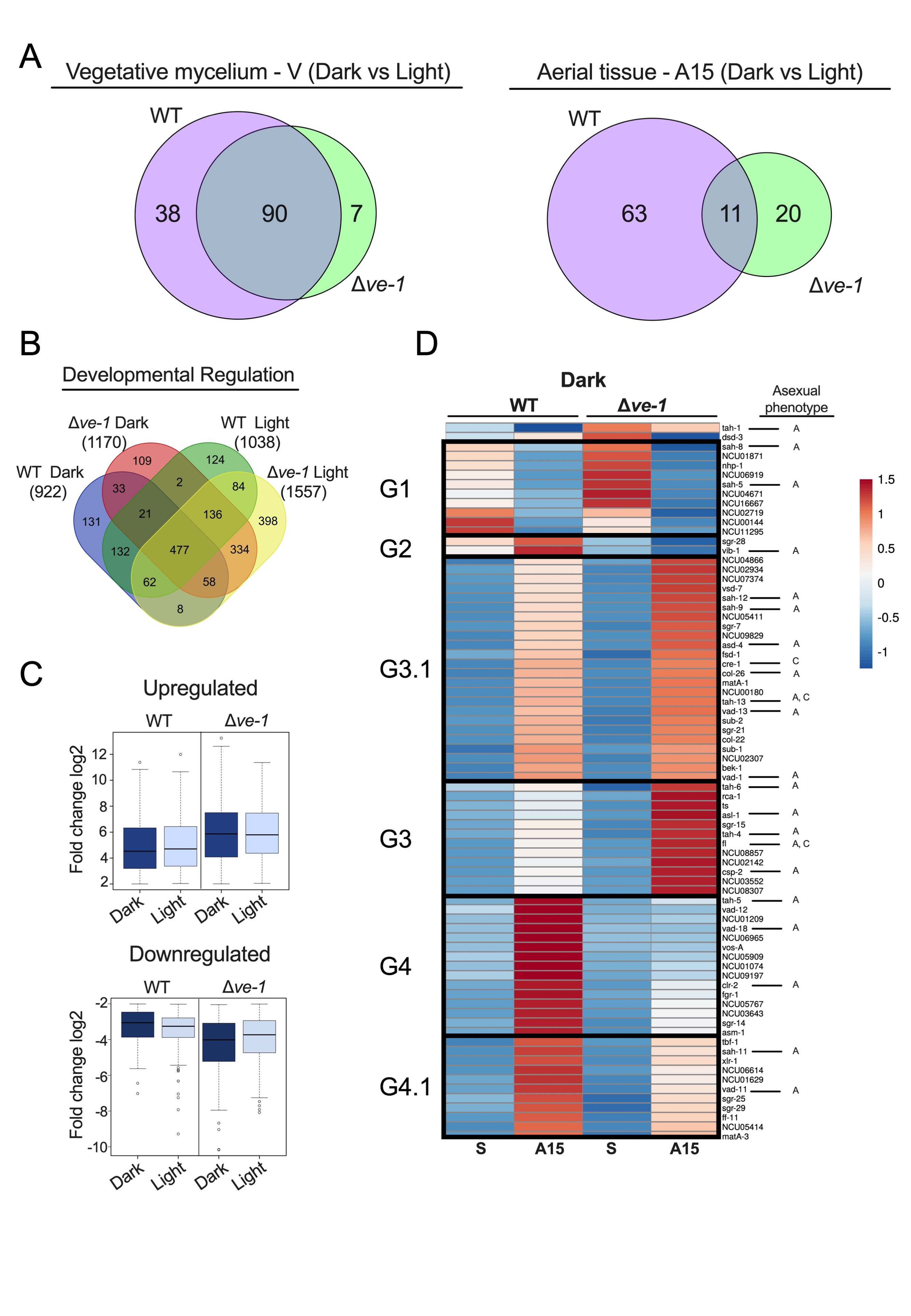

Supplement: FIG S3 [file mbio.01505-22-s0003.jpg]
