# Supplementary material for: Transcriptional Regulation by the Velvet Protein VE-1 during Asexual Development in the Fungus Neurospora crassa
Source: mBio. 2022 Aug 1;13(4):e01505-22. doi: 10.1128/mbio.01505-22 (PMC9426599; doi:10.1128/mbio.01505-22)
Supplement: TABLE S1 [file mbio.01505-22-s0004.pdf]

Table S1. List of strains used in this study

Strain	Genotype	Source
FGSC#2489	4-OR23-1V mat A	FGSC
FGSC#2490	ORS-SL6a mat a	FGSC
FGSC#11401	*ve-1^{KO} mat A*	FGSC
FGSC#11400	*ve-1^{KO} mat a*	FGSC
FGSC #13050	*ve-2^{KO} mat a*	FGSC
SC-Δ*ve-2*	*ve-2^{KO} mat A*	This study
FGSC #13536	*vos-1^{KO} mat A*	FGSC
SC-Δ*vos-1*	*vos-1^{KO} mat a*	This study
FGSC #22596	*lae-1^{KO} mat a*	FGSC
SC-Δ*lae-1*	*lae-1^{KO}* mat A	FGSC
FGSC #11044	*fl^{KO} mat a*	FGSC
FGSC #11308	*vib-1^{KO} mat a*	FGSC
VE-1::3XFLAG	*ve-1^{FLAG}*	Gil-Sánchez et al., 2022
VE-2::3XFLAG	*ve-2^{FLAG}*	Bayram et al., 2019
LAE-1::3XFLAG	*lae-1^{FLAG}*	Bayram et al., 2019
SC- Δ*ve-1* LAE-1::3XFLAG	*ve-1^{KO} lae-1^{FLAG}*	This study
SC- Δ*ve-1*Δ*ve-2*	*ve-1^{KO} ve-2^{KO}*	This study
SC- Δ*ve-1*Δ*vos-1*	*ve-1^{KO} vos-1^{KO}*	This study
SC- Δ*ve-2*Δ*vos-1*	*ve-2^{KO} vos-1^{KO}*	This study
SC- Δ*ve-1*Δ*lae-1*	*ve-1^{KO} lae-1^{KO}*	This study
SC- Δ*ve-2* Δ*lae-1*	*ve-2^{KO} lae-1^{KO}*	This study
SLC- Δ*ve-1*Δ*vib-1*	*ve-1^{KO} vib-1^{KO}*	This study
SC- Δ*ve-2* Δ*vib-1*	*ve-2^{KO} vib-1^{KO}*	This study
SC- Δ*ve-1*Δ*fl*	*ve-1^{KO} fl^{KO}*	This study
SC- Δ*ve-2*Δ*fl*	*ve-2^{KO} fl^{KO}*	This study
