# Supplementary material for: Transcriptional Regulation by the Velvet Protein VE-1 during Asexual Development in the Fungus Neurospora crassa
Source: mBio. 2022 Aug 1;13(4):e01505-22. doi: 10.1128/mbio.01505-22 (PMC9426599; doi:10.1128/mbio.01505-22)
Supplement: TABLE S2 [file mbio.01505-22-s0005.pdf]

Table S2. List of primers used in this study

Primers	Sequence 5'-3'	Use
con10-45F	CAGCCACAGCGGAGGC	RT-PCR
con10-104R	TTGGAGCAATTTCGCGC	RT-PCR
ve1-939F	CGGAGGCCATCTGGGTTT	RT-PCR
ve1-998R	GGTGGTTGAGCGGGATACTG	RT-PCR
ve-2-86F	CGCGTCATCACCAATTGC	RT-PCR
ve-2-187R	ACATGGTGGGATCTTGGTATGG	RT-PCR
vos-1-460F	GCACAGCCCTCAGTCTAACCA	RT-PCR
vos-1-560F	CACTCCGGTACCATGCAACA	RT-PCR
lae-1-33 F	CCAGGACAGTTATCCGTCTTACAGA	RT-PCR
lae-1-133 R	CCCCATAGAAGCGTCCGTTT	RT-PCR
RTcsp-2F	AGGTTCAACTCGAGCGATGCT	RT-PCR
RTcsp-2R	GACTCAATCCAGCCCGTCAA	RT-PCR
acon3-771F	GCAACGTGCCCCTAACTTTG	RT-PCR
acon3-832R	TGGTACCCGCGCAATTTC	RT-PCR
acon-2-76F	TCCGAGGACCGCTGCAT	RT-PCR
acon-2-145R	AGTTTGGTTCCGTCTTTGCAA	RT-PCR
fl-811F	GGCGATTCCCGCTACTGTT	RT-PCR
fl-869R	TTGCAGGCCTTTCCCAAA	RT-PCR
fld-1275F	CGCCAACGCCCAGAAA	RT-PCR
fld-1350R	GCAGCGCATGACAACGAA	RT-PCR
vib-740F	CCACCCAACTCACCAGAAGT	RT-PCR
vib-842R	GTGATCTCCGCCATTGTCTT	RT-PCR
tub-1073F	CCCGCGGTCTCAAGATGT	RT-PCR
tub-1139R	CGCTTGAAGAGCTCCTGGAT	RT-PCR
5'UTR-ve1-sc	TCATACCCGTCGCTCTGGTTTCTGG	PCR
3've1int-sc	GTTGGCTCGTGGGGTAGACGG	PCR
5'UTR-ve1-sc	AACCTGATTATGCCAGGCCGAT	PCR
ve-2-5'F-ORF-sc	ATGCAACCGTACACTTCAAACC	PCR
ve-2-3'R-ORF-sc	CCG CCT TAA TAA TCA TCA TCA TCC	PCR
3'UTRve-2R-sc	CCGCCTGCGCCTTACCTCC	PCR
5've-2intF-sc.	TGGTCAGGGCGGACAGCA	PCR
vos-1-5'F-ORF-sc	ATGGCAACAGCATCGCCGCC	PCR
vos-1-3'R-ORF-sc	GGC CTT ACT TTG GGT CGG C	PCR
5'UTRlaeA-sc	CGA CCG CTA TCT AGC ACG TT	PCR
3'UTRlaeA-sc	TCG GAC AGA ACC CAC CAA AG	PCR
3'lae1int-sc	ACCCAATCGATTTCCACCTGCTC	PCR
hygB-R	CGTGCACGCGGATTTCGGCTCC	PCR
hygB-1-sc	GGTCAATACACTACATGGCGTG	PCR
hygB-2-sc	CACGCCATGTAGTGTATTGACCG	PCR
